# A short report on ADHD detection using convolutional neural networks

**DOI:** 10.3389/fpsyt.2024.1426155

**Published:** 2024-09-05

**Authors:** Vikram Kulkarni, Bhushankumar Nemade, Shreyaskumar Patel, Keyur Patel, Srikanth Velpula

**Affiliations:** ^1^ Department of Information Technology, Mukesh Patel School of Technology Management & Engineering, SVKM’s NMIMS, Mumbai, Maharashtra, India; ^2^ Department of CSE, Shri L R Tiwari College of Engineering, Mumbai, Maharashtra, India; ^3^ Institute of Electrical and Electronics Engineers (IEEE), Dallas, TX, United States; ^4^ Institute of Electrical and Electronics Engineers (IEEE) - Engineering in Medicine and Biology Society, New York, NY, United States; ^5^ Department of EEE, SR University, Warangal, Telangana, India

**Keywords:** deep learning, attention deficit hyperactivity disorder, convolutional neural networks, behavioral patterns, complex neurobiological features, electroencephalogram (EEG), magnetic resonance imaging (MRI)

## 1 Introduction

Attention Deficit Hyperactivity Disorder (ADHD), is characterized by abnormalities in brain structure and function, particularly in the prefrontal cortex, which is associated with attention, executive functions, and impulse control. Neurochemical imbalances, especially involving dopamine and norepinephrine, play a crucial role. Genetic factors contribute significantly, with ADHD often running in families. ADHD affects both children and adults, with symptoms typically manifesting in childhood (1). Those with ADHD may experience a range of abnormalities, including difficulties in maintaining attention, hyperactivity, and impulsivity (2). These symptoms can lead to academic challenges, difficulties in social interactions, and problems with task completion and organization. In adults, ADHD can result in issues with time management, occupational performance, and maintaining relationships. Conventional diagnostic methods often depend on subjective evaluations and standardized surveys, which may result in inconsistencies and incorrect diagnoses (3).

Given these challenges, integrating DL into the diagnosis of ADHD represents a significant advancement, facilitating objective, data-driven approaches that enhance diagnostic precision and clinical decision-making (4). Existing diagnostic strategies encounter difficulties in adequately capturing the broad spectrum of ADHD symptoms, underscoring the necessity for more accurate and tailored methodologies (Sathiya et al., 2024).

Convolutional Neural Networks (CNNs)-based specialized models excel at image recognition tasks. In the case of ADHD, researchers can employ CNN to analyze brain-imaging data, such as functional MRI scans (5). By learning hierarchical features from raw pixel values, CNN automatically detect relevant brain structures and abnormalities associated with ADHD (6). The use of Deep Learning (DL) technology marks the beginning of a new era in neuropsychiatric evaluations. DL models can scrutinize extensive datasets, detecting complex patterns that elude human perception. By utilizing CNN, healthcare providers and researchers may overcome enduring obstacles in ADHD care. These sophisticated analytical instruments enhance diagnostic precision, potentially forecasting the efficacy of therapeutic interventions. As technology and healthcare intersect, we can envision a future where personalized ADHD assessments better cater to those impacted by this condition (7).

The updated 2D block diagram illustrates the central role of Convolutional Neural Networks (CNN) in the detection and analysis of ADHD (Attention Deficit Hyperactivity Disorder) by integrating various data sources and influences. The central circle represents the CNN, surrounded by peripheral circles labeled Voice Signals, Brain Images, Behavioral Data, EEG Signals, fMRI Data, Genetic Data, External Influences, and ADHD Init. Each peripheral circle is connected to the CNN, indicating the flow of data from these sources into the neural network for comprehensive analysis. Voice Signals provide insights into behavioral and neurological patterns, while Brain Images and fMRI Data reveal structural and functional brain abnormalities. Behavioral Data and EEG Signals offer information on symptom severity and brain activity, respectively. Genetic Data highlights hereditary aspects, and External Influences encompass prenatal factors, early childhood environment, family dynamics, educational and social settings, and socioeconomic status. The ADHD Init represents the initial diagnosis, serving as the starting point for further analysis. This integration allows the CNN to provide a detailed and accurate detection of ADHD by analyzing multiple dimensions of data, emphasizing a multi-faceted approach to improving diagnostic accuracy and understanding the disorder.

## 2 Role of CNN in ADHD diagnosis

CNN, a form of deep learning, have demonstrated impressive capabilities in image processing. Through convolution operations, CNN detect and extract local features from images, combining these to form higher-level features. This ability makes CNN particularly effective for classifying images by extracting pixel values and their feature vectors, enhancing the network’s comprehension and resulting in precise classification. Consequently, researchers increasingly utilize CNN to investigate brain, mental, and neurological disorders (8).

In studying ADHD, CNN are favored for their exceptional ability to handle spatial hierarchies and extract relevant features from neuroimaging data like MRI or fMRI scans (9). CNN can effectively capture spatial relationships within data, crucial for brain scans where the positioning and interaction of different brain regions are significant (10). The convolutional layers in CNN automatically learn to detect important features such as edges, textures, and patterns, enabling the identification of abnormalities or patterns associated with ADHD (11). Moreover, CNN are computationally efficient, using parameter sharing and pooling layers to reduce the number of parameters and the dimensionality of the data. This reduction in complexity allows CNN to handle high-dimensional data, such as 3D brain scans, more efficiently than fully connected networks (12).

The robustness of CNN to variations, such as shifts and distortions in input data, adds to their suitability for neuroimaging studies. This robustness is crucial when dealing with biological data with inherent variability. CNN have a proven track record of superior performance in a wide range of image-based tasks, from object detection to medical image analysis, which translates well to neuroimaging tasks (13).

Advanced CNN techniques, such as 3D CNN, are particularly effective for volumetric data like MRI, as they can capture the 3D spatial structure of the brain more accurately than traditional 2D CNN or other deep learning models. Transfer learning further enhances CNN performance by leveraging pre-trained models on large image datasets, which can be fine-tuned for specific ADHD-related tasks, requiring less training data while achieving high accuracy (14). Additionally, CNN offer interpretable visualizations through techniques like Grad-CAM (Gradient-weighted Class Activation Mapping), allowing researchers to see which parts of the brain contribute most to the network’s decisions. This interpretability aids in understanding and trusting the results, making CNN a preferred choice in ADHD research over other deep learning algorithms (15).

The study (16) utilizes EEG signals from children with ADHD and healthy peers, recorded during a task. After pre-processing, the data is segmented, and frequency features are extracted and fed into a CNN. The Layer-wise Relevance Propagation (LRP) algorithm is used to identify and select the most relevant channels for classification. The proposed method achieved an accuracy of 94.52% for validation data. The study demonstrates that the method can effectively diagnose ADHD and provides insights into the importance of specific brain regions and frequency bands, particularly the gamma II band in the frontal and central regions, which are significant for higher-order neurocognitive processes.

The paper introduces the Frequency-Integrated Visual-Language Network (FIVLNet), a DL framework designed to enhance diagnostic accuracy for ADHD using MRI scans. Traditional DL methods often fail to capture the sequential dependencies and complex structural details of MRI images, leading to lower classification accuracy. FIVLNet addresses this by combining high and low-frequency data from MRI images through a CNN and cross-attention mechanism, resulting in more comprehensive image representations. Additionally, it incorporates textual embeddings from Contrastive Language-Image Pre-training (CLIP) to enrich the model’s learning capacity. Despite these enhancements, FIVLNet maintains a lightweight architecture with fewer learnable parameters compared to existing models. FIVLNet achieves an accuracy of 93.89% on fMRI data (17).

A study by Dubreuil-Vall, Ruffini, and Camprodon demonstrated that DL CNN can effectively differentiate between adults with ADHD and healthy individuals using event-related spectral EEG data. The CNN model, trained on spectrograms from the Flanker Task, achieved an 88% classification accuracy, outperforming traditional neural networks. The key findings indicated decreased alpha band power and increased delta-theta band power in ADHD patients, highlighting potential biomarkers for ADHD diagnosis. This research underscores the promise of DL in developing clinically useful diagnostic tools (18).

The article (19) discusses the implementation of a DL model for detecting ADHD using ECG signals. The proposed model utilizes a one-dimensional CNN comprising convolutional, pooling, and fully connected layers. It employs techniques like dropout, ReLU activation, and L2 kernel regularization to mitigate overfitting. The model was trained with the Adam optimizer and utilized weighted loss to address data imbalance. The model achieved significant feature reduction and accurate classification, highlighting the potential of ECG-based DL methods in ADHD diagnosis.

The research article (20) explores the application of CNN to classify ADHD in children using functional Magnetic Resonance Imaging (fMRI) data. The study tests three models—Nadam, SGDM, and a proposed CNN—on fMRI datasets, finding that the proposed CNN achieves the highest accuracy at 98.77%. This superior performance underscores the potential of CNN in providing accurate ADHD diagnosis, suggesting that DL techniques can be effectively utilized for early and precise detection of ADHD, thus aiding in timely intervention and treatment.

The researchers zou et al. in (21) developed a 3D CNN based DL model for classifying ADHD using MRI scans. They extracted 3D low-level features from fMRI and sMRI scans and designed a multimodality CNN architecture to combine them. This approach yielded an accuracy of 69.15%.

The work proposed in (22) utilized the ADHD-200 dataset to diagnose ADHD. They trained a deep multimodal 3D CNN from features obtained from gray matter and fALFF from fMRI. Then output scores were classified with KNN, SVM and LDA algorithms. LDA showed better results among the three classifiers, with a classification accuracy of 74.93%.

The paper (23) presents a novel ADHD classification method combining Convolutional Denoising Autoencoders (CDAE) and Random Forest (RF) algorithms, demonstrating superior performance on the ADHD-200 dataset. The proposed approach extracts deep spatio-temporal features from fMRI data, achieving higher accuracy (75.64%), sensitivity (76.922%), and specificity (73.08%) compared to traditional methods like MKL, MDA-SVM, and 3D-CNN. The research highlights the effectiveness of ensemble learning and grid search optimization for hyperparameter tuning. Future work aims to expand datasets, explore additional feature extraction techniques, and enhance model interpretability to further improve ADHD classification and facilitate clinical applications.

This study (24) introduces the RBP-CNN model, a convolutional neural network designed for precise brain tumor classification in medical imaging. It incorporates regional binary patterns (RBP) and Gray Standard Normalization (GSN) preprocessing to address challenges in extracting image noise and texture features. The model achieves a classification accuracy of 96% with a 7% false classification rate on a dataset of 3000 samples. RBP-CNN’s novel approach and superior performance make it a potential state-of-the-art tool for medical image analysis, demonstrating robustness and scalability on the FigShare dataset. This research provides a new methodology for future exploration in hyperspectral image applications.

A comprehensive search was conducted for English articles on sMRI or/and fMRI-based machine learning techniques for diagnosing ADHD until March 2024. Diagnostic value was assessed by calculating pooled sensitivity, specificity, positive and negative likelihood ratios, and area under the curve (AUC). Heterogeneity was examined using the I2 test and meta-regression analysis, while publication bias was assessed with the Deeks funnel plot asymmetry test. The systematic review included 43 studies, with 27 included in the meta-analysis. The pooled sensitivity and specificity of sMRI or/and fMRI-based ML techniques were 0.74 and 0.75, respectively. The AUC was 0.81, indicating relatively good diagnostic value for ADHD. However, the meta-analysis focused solely on sMRI or/and fMRI-based ML techniques, excluding EEG-based methods, suggesting the need for further analyses on multimodal medical data. In conclusion, sMRI or/and fMRI-based ML techniques show promise as objective diagnostic methods for ADHD (25). The insights, methods used, results, and limitations of recent studies are summarized in the following **Table 1**.

**Table 1:** Analysis of various methods in ADHD detection using CNNs.

Ref. No	Insights	Methods Used	Results	Limitations
(16)	Effective ADHD diagnosis using EEG signals; importance of specific brain regions and frequency bands identified	EEG signals, preprocessing, frequency feature extraction, CNN, Layer-wise Relevance Propagation	94.52% accuracy	Focused only on EEG data; small sample size
(17)	Enhanced diagnostic accuracy using FIVLNet framework combining MRI image data	FIVLNet, high and low-frequency data, CNN, cross-attention mechanism, textual embeddings (CLIP)	93.89% accuracy on fMRI data	Focuses on MRI data; does not address multimodal data
(18)	Differentiated adults with ADHD from healthy individuals using event-related spectral EEG data	Event-related spectral EEG data, CNN	88% classification accuracy	Limited to spectral EEG data; specific to adult population
(19)	ECG-based DL model for ADHD detection	One-dimensional CNN, dropout, ReLU activation, L2 kernel regularization, Adam optimizer	Significant feature reduction, accurate classification	Limited to ECG data; potential overfitting not extensively tested
(20)	High accuracy in ADHD diagnosis using fMRI data with different models	fMRI data, Nadam, SGDM, proposed CNN	Proposed CNN: 98.77% accuracy	Specific to fMRI data; does not explore multimodal data
(21)	Developed 3D CNN for classifying ADHD using MRI scans	3D CNN, multimodality architecture combining fMRI and sMRI data	69.15% accuracy	Lower accuracy compared to other methods
(22)	Diagnosed ADHD using deep multimodal 3D CNN with gray matter and fALFF features	ADHD-200 dataset, deep multimodal 3D CNN, KNN, SVM, LDA	LDA: 74.93% accuracy	Only uses ADHD-200 dataset; compares limited classifiers
(23)	Combined CDAE and RF for superior ADHD classification	Convolutional Denoising Autoencoders, Random Forest, ensemble learning, grid search optimization	75.64% accuracy, 76.922% sensitivity, 73.08% specificity	Needs larger datasets; explores limited feature extraction techniques
(24)	Precise brain tumor classification using RBP and GSN preprocessing	RBP, Gray Standard Normalization (GSN), CNN	96% accuracy, 7% false classification rate on a dataset of 3000 samples	Specific to brain tumor classification; does not address ADHD
(25)	Comprehensive review and meta-analysis of sMRI and fMRI-based ML techniques for ADHD diagnosis	sMRI, fMRI, ML techniques, sensitivity, specificity, likelihood ratios, AUC, meta-regression	Sensitivity: 0.74, Specificity: 0.75, AUC: 0.81	Excludes EEG-based methods; needs further analyses on multimodal data
(26)	Precise brain tumor classification using RBP and GSN preprocessing	RBP, Gray Standard Normalization (GSN), CNN	96% accuracy, 7% false classification rate on a dataset of 3000 samples	Specific to brain tumor classification; does not address ADHD
(27)	Comprehensive review and meta-analysis of sMRI and fMRI-based ML techniques for ADHD diagnosis	sMRI, fMRI, ML techniques, sensitivity, specificity, likelihood ratios, AUC, meta-regression	Sensitivity: 0.74, Specificity: 0.75, AUC: 0.81	Excludes EEG-based methods; needs further analyses on multimodal data

DL approaches have brought significant advancements in the diagnosis and management of ADHD, marking a substantial improvement over traditional methods. This study focuses on advanced CNN to provide a detailed analysis of their application in ADHD detection. CNN analyze various data sources, such as behavioral patterns, identifying complex features associated with ADHD. For instance, methods utilizing EEG signals have achieved 94.52% accuracy, while those employing fMRI data have reported up to 98.77% accuracy, showcasing the effectiveness of CNN in handling diverse data types (16) (20). Integrating these approaches can enhance diagnostic precision and develop customized treatments, improving efficacy. This study highlights the revolutionary potential of CNN in ADHD diagnosis and care, opening the path for more effective and personalized treatment.

## 3 Future directions

Future research directions encompass the development of AI models that are explainable, the integration of real-time monitoring tools, and the expansion of collaborative networks for data exchange and validation. Moreover, it is imperative to establish ethical frameworks and regulatory requirements to ensure the responsible utilization of DL technology in clinical settings. Cutting-edge methodologies, like meta-learning and model distillation, present promise in improving model interpretability and transparency, thereby promoting trust and acceptance among healthcare providers and patients.

To address these challenges, future research should focus on the following areas:

1. Combining Neuroimaging with Behavioral Data: Investigate the integration of MRI/fMRI scans with behavioral data to enhance diagnostic accuracy. This could involve developing models that can process and learn from both image and text data simultaneously.
2. Incorporation of EEG and ECG Data: Expand the research to include other physiological data such as EEG and ECG, which can provide complementary information about brain activity and cardiac function, respectively.
3. Exploration of 3D CNN: Further explore the use of 3D CNN for volumetric neuroimaging data to capture the 3D spatial structure of the brain more accurately.
4. Hybrid Models: Develop hybrid models that combine CNN with other DL architectures like LSTM or transformers to capture temporal dynamics and sequential dependencies in neuroimaging data.
5. Prediction of Treatment Outcomes: Use CNN to predict the efficacy of various therapeutic interventions based on individual neuroimaging and behavioral profiles. This can help in creating personalized treatment plans for ADHD patients.
6. Longitudinal Studies: Conduct longitudinal studies to track changes in brain patterns over time with different treatments, helping to refine and personalize therapeutic approaches.
7. Focus on Explainability: Develop methods to enhance the interpretability of CNN models, such as using techniques like Grad-CAM to visualize which brain regions contribute most to the diagnosis. This can help clinicians trust and understand the model’s decisions.
8. Deployment in Clinical Settings: Work on translating these advanced CNN models into practical tools that can be used in clinical settings. This involves addressing challenges related to scalability, real-time processing, and integration with existing healthcare systems.

These future directions aim to enhance the precision, reliability, and applicability of CNN-based approaches in diagnosing and treating ADHD, ultimately improving patient outcomes and advancing the field of neuropsychiatric research.

## 4 Conclusion

This study demonstrates the transformative potential of CNNs in ADHD diagnosis and treatment, offering significant improvements over traditional methods. CNNs provide an objective, data-driven approach that enhances diagnostic precision and clinical decision-making by analyzing complex neurobiological features from various data sources. Future research should focus on integrating multimodal data and developing personalized treatment plans while ensuring ethical considerations and explainable AI models. In conclusion, CNNs represent a paradigm shift in ADHD care, paving the way for more precise, personalized, and effective treatments, with continuous research promising significant improvements in patient quality of life.
